# Unbiased transcriptome analysis of human cleft palate reveals evolutionally conserved molecular signatures of development: experimental study

**DOI:** 10.1097/JS9.0000000000001841

**Published:** 2024-06-21

**Authors:** Taehee Jo, Jeonghoon Kim, Jaehoon Choi, Junhyung Kim, Woonhyeok Jeong

**Affiliations:** Department of Plastic and Reconstructive Surgery, Dongsan Medical Center, Keimyung University College of Medicine, Daegu, Korea

**Keywords:** cleft palate, embryology, epigenesis, genetic, nontherapeutic human experimentation, RNA, sequence analysis

## Abstract

**Background:**

The development of the secondary palate, an essential process for hard palate formation, involves intricate cellular processes. Here, the authors examined the expression patterns of palatal fusion-associated genes in postdevelopmental human palatal tissues.

**Methods:**

Mucosal samples collected from the anterior fused (control; *n*=5) and posterior unfused regions (study; *n*=5) of cleft palate patients were subjected to RNA sequencing. Gene Set Enrichment Analysis (GSEA) was conducted to identify consistent changes in molecular signaling pathways using hallmark (h) gene set collections from the Molecular Signature Database v7.4. The results of RNA sequencing were validated by epithelial-mesenchymal transition (EMT) assays with suppression of target genes, including *lrp6, shh, Tgfβ-3* (Bioneer), and negative control siRNA in a human fibroblast cell line (hs68).

**Results:**

Transcriptome profiling of the cleft mucosa demonstrated that the fully fused anterior mucosa exhibited globally upregulated EMT, Wnt β-catenin, Hedgehog, and TGF-β signaling pathways in gene set enrichment. This strongly indicates the evolutionary conserved similarities in pathways implicated in palatogenesis, as previously shown in murine models. In EMT assays with suppression of Lrp6, Shh, and TGF-β3 in human fibroblast cell lines, suppression of Lrp6 exhibited consistent suppression effects on EMT markers. This indicates a closer association with EMT compared to the other two signals.

**Conclusion:**

Our study highlights evolutionarily conserved molecular signatures and provides insights into the importance of the EMT pathway in palatal fusion in humans. Furthermore, intraindividual comparative analysis showed the spatial regulation of gene expression within the same organism. Further research and animal models are needed to explore the complexities of EMT-related palatal fusion.

## Introduction

Highlights

- Transcriptome analysis reveals conserved epithelial-mesenchymal transition (EMT), Wnt, Hedgehog, and TGF-β pathways in human cleft palate.
- Comparative analysis of fused and nonfused mucosa shows spatial regulation of gene expression.
- Findings support the evolutionary conservation of molecular mechanisms in palatal fusion.
- Lrp6 gene suppression in fibroblasts highlights its significant role in EMT pathway regulation.

Cleft palate is one of the most common craniofacial birth defects in humans^1,2^. The palate is crucial for sucking, swallowing, and normal speech production, with the ability to divide the oral cavity and nasal cavity^3^. Cleft palate is the result of defective palatogenesis, and it is thought to be caused by multiple environmental or genetic factors^4,5^. Cleft palate can manifest as either complete or incomplete, giving rise to a range of diverse combinations^1,6–8^. The secondary palate represents the posterior extension from the primary palate, ultimately forming the hard and soft palates^4^. The development of the secondary palate commences within the palatal shelves, which initially assume a vertically positioned configuration on both sides of the tongue^4^. Between the seventh and eighth weeks of embryonic development, concurrent with mandibular growth, the palatal shelves undergo caudal movement along with the tongue, transitioning to a horizontal orientation above the tongue^9^. The inner epithelial border of the palatal shelves is referred to as the medial edge epithelium (MEE), and the convergence of the bilateral MEE at the midline results in the formation of the midline epithelial seam (MES)^9–11^. Eventually, the epithelial cells constituting the midline epithelial seam undergo apoptosis, being replaced by mesenchyme and culminating in the formation of the secondary palate^10^.

The term employed for this phenomenon is palatal fusion, and numerous investigations have been conducted concerning the genetic factors implicated in palatal fusion^4,10–13^. However, the majority of these studies have primarily relied on mouse embryo models, with a dearth of research employing actual human tissue samples. RNA sequencing (RNA-Seq) is a powerful transcriptomic technique that uses next-generation sequencing (NGS) technology to profile and quantify the entire transcriptome^14^. These capabilities enable the acquisition of comprehensive data on entire transcripts at a particular location and time, facilitating the analysis of transcriptomic variations while eliminating bias. Furthermore, it is feasible to examine individual genes that show significant upregulation or downregulation to ascertain their association with a specific signaling pathway through gene set enrichment analysis (GSEA) with RNA-Seq data^15^. Instead of focusing on individual gene expression changes, GSEA considers aggregate expression changes within predefined gene sets, thus providing insights into the underlying biological processes affected by the condition under study.

Therefore, the researchers of this study attempted to identify disparities in the expression patterns of palatal fusion-associated genes, as previously observed in murine models, within postdevelopmental human palatal tissue through RNA-seq analysis.

## Methods

### Sampling and RNA extraction

**Figure 1:**
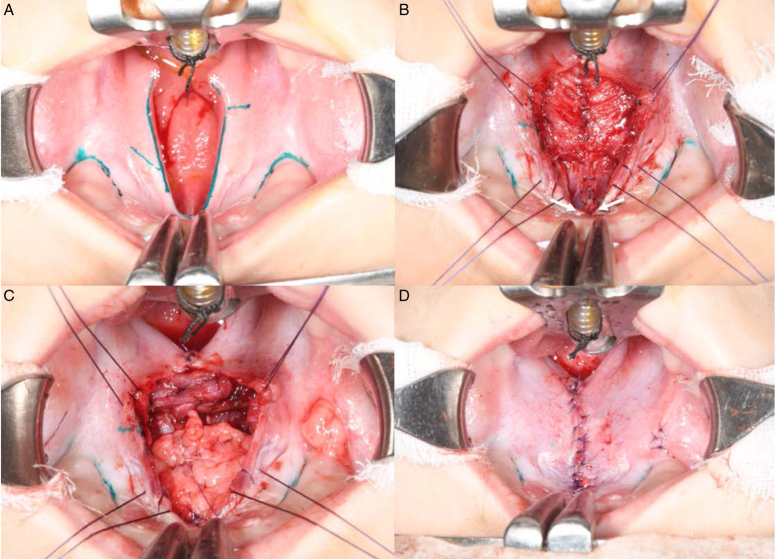
Harvest of mucosal tissue (A) The nonfused posterior mucosa was collected from the uvula (*white asterisk*). (B) The fused anterior mucosa was harvested from the oral-side mucosal flap immediately anterior to the cleft palate after completing the elevation of the oral-side mucosal flap (*white arrow*). To prevent excessive tension during mucosal flap closure, the flap was harvested within a width of 1 mm. (C) Depiction of the procedure of overlapping intravelar veloplasty and buccal fat pad flap. (D) Immediate postoperative appearance.

This study enrolled five consecutive patients who met the inclusion criteria among those with cleft palate who visited our institution between January 2021 and June 2021 (Table 1). The inclusion criteria were nonsyndromic cleft palate and incomplete cleft palate. The exclusion criteria were patients with syndromic cleft palate, adult patients, secondary surgery cases, and patients with complete cleft palate without any fused palatal region. The guardians fully understood the purpose and methods of the study and agreed to participate according to the protocol approved by the Institutional Review Board (2020-07-034). During the operation, mucosal samples were harvested from fused anterior (control group) and unfused posterior (study group) parts of cleft palates (Fig. 1). The specimens from the anterior served as the control, while the specimens from the posterior were regarded as the cleft study groups. In palatal fusion, the region playing a crucial role is the MEE of the palatal shelves^16^. The anatomical location of MEE corresponds to the mucosa of the cleft margin in patients after birth. In this experiment, the control group consisted of fused mucosa harvested from the midline immediately in front of the anterior limit of the cleft palate, while the study group used the medial edge mucosa of the always unfused uvula from patients. Each sample was immediately frozen in liquid nitrogen. The tissues were then homogenized with 1 ml of TRIzol reagent (Invitrogen) in independent bead-containing safe lock tubes. After TRIzol/chloroform extraction, the aqueous phase was mixed with 100% EtOH and then loaded onto an RNeasy column (Qiagen). The RNA extraction process was continued according to the manufacturer’s protocol.

**Table 1:** Characteristics of palates.

Patient No.	Sex	Age	Type of cleft palate	Medical history	Surgical procedure
1	M	13 mo	Veau class I	ASD, accessory spleen, microcephaly	Double opposing Z-plasty
2	M	12 mo	Veau class I	RDS	Double opposing Z-plasty
3	M	26 mo	Veau class II	None	Sommerlad’s palatoplasty
4	F	12 mo	Veau class II	None	Sommerlad’s palatoplasty
5	M	10 mo	Veau class II	Hypothyroidism, plagiocephaly, ASD	Sommerlad’s palatoplasty

### RNA-sequencing data analysis

Control samples (*n*=5) and cleft study samples (*n*=5) were subjected to analysis. For RNA sequencing analysis, the libraries were prepared for 151 bp paired-end sequencing using a TruSeq stranded mRNA Sample Preparation Kit (Illumina). Namely, mRNA molecules were purified and fragmented from 1 μg of total RNA using oligo (dT) magnetic beads. The fragmented mRNAs were synthesized as single-stranded cDNAs by random hexamer priming. By applying this as a template for second-strand synthesis, double-stranded cDNA was prepared. After the sequential process of end repair, A-tailing, and adapter ligation, cDNA libraries were amplified with PCR. The quality of these cDNA libraries was evaluated with the Agilent 2100 BioAnalyzer (Agilent). They were quantified with the KAPA library quantification kit (Kapa Biosystems) according to the manufacturer’s library quantification protocol. Following cluster amplification of denatured templates, paired-end (2×151 bp) sequencing was performed using an Illumina NovaSeq6000 (Illumina). The adapter sequences and the ends of the reads less than Phred quality score 20 were trimmed, and reads shorter than 50 bp were simultaneously removed by using cutadapt v.2.8. Filtered reads were mapped to the reference genome related to the species using the aligner STAR v.2.7.1a following ENCODE standard options with the ‘-quantMode TranscriptomeSAM’ option for estimation of transcriptome expression level^17^. Gene expression estimation was performed by RSEM v.1.3.1^18^. To normalize sequencing depth among samples, transcript per million (TPM) values were calculated. The TPM values were then processed for principal component analysis (PCA) and unsupervised hierarchically clustered heatmap generation. Gene set enrichment analysis (GSEA) was performed with hallmark (h) gene set collections of the Molecular Signature Database v7.4^15^. FDR q <0.15 was considered significant.

Cellular epithelial-mesenchymal transition (EMT) assay

The results of RNA sequencing were validated by EMT assays with suppression of target genes, including *lrp6, shh,* and *Tgfβ-3* ^19,20^. The human fibroblast cell line (hs68) was maintained in Dulbecco’s modified Eagle’s medium (DMEM) supplemented with 10% fetal bovine serum, penicillin (100 U/ml), and streptomycin (100 mg/ml). The cells were transfected with siRNAs using Lipofectamine RNAiMAX (Invitrogen) according to the manufacturer’s protocol. Predesigned siRNAs targeting the following genes were purchased and used: *lrp6, shh,* and *Tgfβ-3* (Bioneer), and negative control siRNA (5’-GTCAAACGCCTTGTATTTA-3’). Cells were harvested 48 h after transfection. Total RNA was extracted using a TRIzol RNA extraction kit (Invitrogen) according to the manufacturer’s protocol. Total RNA was reverse transcribed to cDNA using an iScript cDNA synthesis kit (Bio-Rad). Then, quantitative real-time PCR was performed using iTaq Universal SYBR Green Supermix (Bio-Rad Bio-Rad) and a CFX Connect Thermal Cycler (Bio-Rad).

*36b4* was used as a reference gene, and the results are presented as relative expression to the control. PCR primers (OriGene Technologies, Inc.) were used as commercially available ones, and the list is as follows (5’-3’): *36b4* (forward), TGG TCA TCC AGC AGG TGT TCG A; *36b4* (reverse), ACA GAC ACT GGC AAC ATT GCG G; *col1a1* (forward), GAT TCC CTG GAC CTA AAG GTG C; *col1a1* (reverse), AGC CTC TCC ATC TTT GCC AGC A; *fibronectin* (forward), ACA ACA CCG AGG TGA CTG AGA C; *fibronectin* (reverse), GGA CAC AAC GAT GCT TCC TGA G; *n-cad* (forward), CCT CCA GAG TTT ACT GCC ATG AC; *n-cad* (reverse), GTA GGA TCT CCG CCA CTG ATT C; *fsp-1* (forward), CAG AAC TAA AGG AGC TGC TGA CC; *fsp-1* (reverse), CTT GGA AGT CCA CCT CGT TGT C; *vimentin* (forward), AGG CAA AGC AGG AGT CCA CTG A; and *vimentin* (reverse), ATC TGG CGT TCC AGG GAC TCA T.

### Statistical analysis

The data are presented as the mean±standard error of the mean (SEM). Statistical differences were compared using a two-tailed, unpaired *t-test*, and differences with *P*<0.05 were considered statistically significant.

## Results

### Posterior and anterior mucosal samples exhibited differentially expressed gene profiles

**Figure 2:**
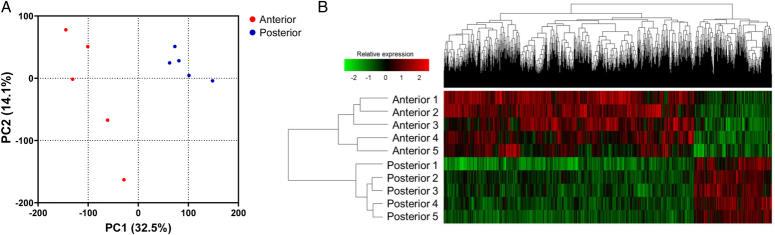
Principal component analysis (PCA) and hierarchically clustered heatmap. (A) The PCA plot displayed noticeable distinctions between the transcriptomes of the posterior study and the anterior control groups. (B) The heatmap demonstrates the hierarchical clustering of the transcriptomes between the study and control samples.

Ten mucosal samples were subjected to RNA-seq analysis: the (1) anterior control (*n*=5) and (2) posterior study (*n*=5) samples. The PCA plot, which demonstrates sample clustering based on similarity, revealed global differences in the transcriptomes of the posterior study and control samples. The transcriptomes of the posterior samples clustered with each other in the group (Fig. 2). The heatmap also demonstrated hierarchical clustering of the transcriptomes of the samples (Fig. 2). PCA and heatmap results demonstrated that the transcriptome changes resulting from grafting were biologically consistent, allowing for further transcriptomic analysis.

### The human cleft palate transcriptome revealed evolutionally conserved molecular signatures

**Figure 3:**
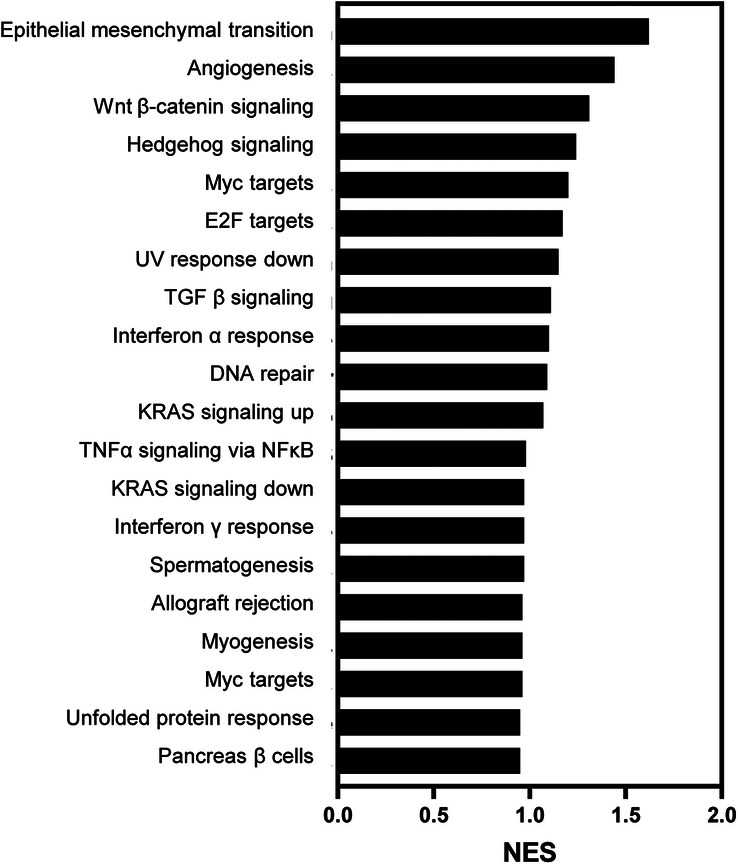
Gene set enrichment analysis (GSEA). In the GSEA results, the most significantly upregulated signaling pathway in the study group was found to be the EMT signaling pathway, which is known to be crucial in cleft palate fusion. Additionally, among the top 10 pathways, the Wnt β-catenin, Hedgehog, and TGF-β signaling pathways, which are also associated with EMT, were included.

**Figure 4:**
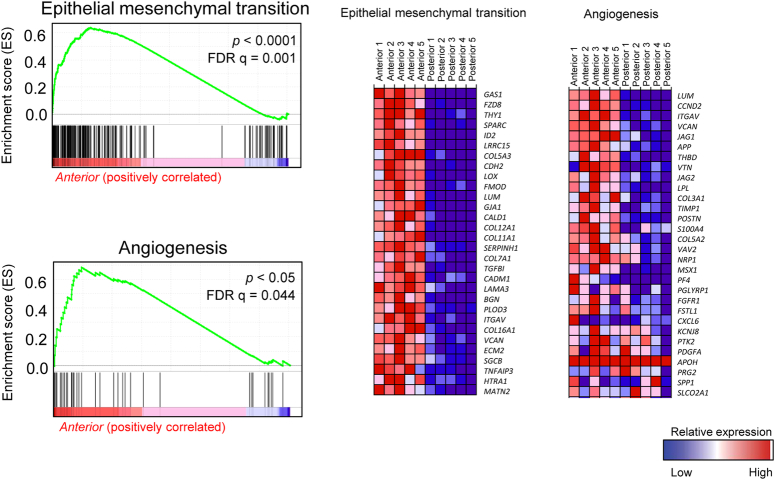
EMT and angiogenesis pathways. EMT and angiogenesis pathways were upregulated in the anterior mucosa in GSEA.

**Figure 5:**
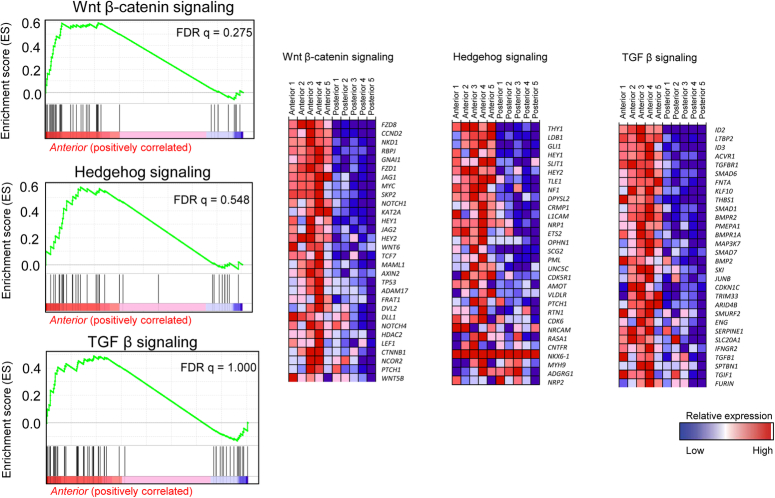
Wnt β-catenin, Hedgehog, and TGF-β signaling pathways. Wnt β-catenin, Hedgehog, and TGF-β signaling pathways, which are the major known molecular pathways of murine cleft palate development, were downregulated in the posterior palatal mucosa.

GSEA, a knowledge-based gene set analysis, identified biologically meaningful gene expression differences among the study groups. We analyzed the transcriptomes of the anterior control group by comparing them with those of the posterior study group. The top-upregulated gene sets in the anterior mucosa were related to epithelial-mesenchymal transition (*FDR q=0.001*) and angiogenesis (*FDR q=0.044*) (Figs. 3 and 4). Regarding the molecular signaling pathways, *Wnt β-catenin* signaling (*FDR q=0.275*), *Hedgehog s*ignaling (*FDR q=0.548*), and *TGF- β* signaling (*FDR q=1.000*) were downregulated in the posterior palatal mucosa (Fig. 5); these are the major known molecular pathways of murine cleft palate development.

### The Lrp6 pathway regulates epithelial-mesenchymal transition-related genes in a human fibroblast cell line

**Figure 6:**
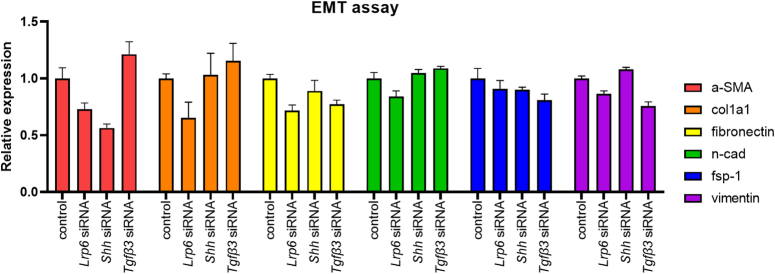
EMT assay. When Lrp6, shh, and Tfgβ3 were inhibited in human keratinocytes using siRNA, only Lrp6 consistently suppressed the EMT pathway. This suggests that in humans, the Lrp6 pathway may more directly affect the epithelial-mesenchymal transition.

Next, to determine the effect of three relevant pathways on EMT in human cell lines, we performed a human cell line study. In hsp67 cells, which are a human fibroblast cell line, we knocked down the *lrp6, shh, and tgf-β3* genes, which directly affect the *Wnt β-catenin, Hedgehog, and TGF- β* signaling pathways, respectively. We then performed real-time PCR of EMT marker genes. As a result, the *lrp6* gene exhibited consistent and decreased expression of all markers of EMT. However, the *shh* and *tgf-β3* genes did not exhibit consistent effects. This suggests that in humans, the Lrp6 pathway may more directly affect the EMT (Fig. 6).

## Discussion

Our research is pioneering in distinguishing gene expression differences between mucosa taken from the cleft margin with high precision during surgery and fused palatal mucosa. We also discovered links to Wnt/β-catenin signaling, impacting EMT. This underscores the genetic pathways shared across species in palatal development, further evidenced by the notable influence of Lrp6 on EMT markers in human fibroblast assays compared to Shh and TGF-β3.

To date, the majority of research on cleft palate formation has predominantly utilized mouse models. Mice exhibit a developmental process that shares similarities with humans, and their relatively short gestation period enables the study of developmental processes within a condensed timeframe. Furthermore, extensive molecular mechanisms have been revealed through previous investigations, rendering mouse models advantageous for research. However, conducting developmental studies on the human population presents ethical concerns, particularly in the context of human palatal development. Consequently, studies on humans have predominantly focused on the investigation of genetic abnormalities commonly observed in large cohorts, revealing a diverse array of genes associated with palatal development^21–24^. Although studies aiming to identify such gene mutations have been conducted successfully, the inherent characteristics of the research methodology pose limitations in attributing disease occurrence rates solely to gene mutations. Furthermore, there are inherent limitations in explaining the diverse phenotypic manifestations of the diseases themselves^25^. Therefore, researchers have conducted studies utilizing the palatal tissues of patients, hypothesizing that the observed changes within the palatal tissues would exhibit a strong association with phenotypic characteristics.

Indeed, alterations in gene function can manifest without modifications to the DNA sequence, referred to as epigenetic regulation. Epigenetic regulation, including DNA methylation, histone modifications, and microRNA, contributes to the process of epigenetic inheritance^21^. Epigenetic features, such as DNA methylation, are maintained even after development is completed, and previous studies have analyzed DNA methylation in the blood and tissues of individuals with cleft palate^25–28^. It is well-established that DNA methylation represses transcription^29^. We also know that DNA methylation can endure beyond cellular division and exhibit transgenerational heritability. In 2017, Sharp *et al*.^26^ identified DNA methylation patterns within genes of interest, such as TBX1, COL11A2, HOXA2, and PDFRA, which are implicated in palate development across distinct subtypes of cleft palate in blood specimens. Notably, an association between DNA methylation patterns in blood and those in lip tissue has also been established. Consequently, we became interested in the potential disparities in mRNA expression levels within the palatal tissues, given the persistent nature of DNA methylation. To address this query, we employed an unbiased technique known as RNA sequencing to compare the transcriptome profiles of the cleft mucosa and the mucosa immediately anterior to the cleft palate that is fully fused.

Several theories have been proposed to explain the development of cleft lip and palate, such as inadequate migration and proliferation of mesenchymal tissue due to enzymatic activity in the amniotic fluid and an inherent metabolic myopathy of the orbicularis oris muscle^30,31^. Especially, EMT plays an important role in palatal fusion, along with various phenomena occurring in MEE cells related to palatal fusion, such as cell apoptosis and migration^10^. This fundamental cellular phenomenon was initially elucidated by Elizabeth Hay^32^. EMT is a biological process characterized by the phenotypic transformation of epithelial cells into mesenchymal cells, accompanied by alterations in morphology, cytoskeleton, adhesion, and migration capacity^33^. The fusion of MEE cells results in the formation of MES, which ultimately undergoes the process of EMT, transitioning into mesenchymal cells. Evidence supporting this phenomenon includes the use of fluorescent dyes, such as DiI and CCFES, to track MEE cells. MEE cells labeled with these dyes prior to palatal fusion continue to exist in the mesenchyme even after palatal fusion has occurred^10,12^. Our research revealed a statistically significant attenuation of the EMT signaling pathway in the nonfused cleft mucosa, in contrast to the fully fused anterior mucosa. Moreover, a discernible diminishing trend was observed in the cleft mucosa concerning the EMT-associated factors Wnt β-catenin and TGF-β compared to the control group. Interestingly, Alvizi *et al*.^25^ also reported that DNA methylation exhibited a prominent elevation of the EMT pathway, ranking it as the most significantly enriched among the top five pathways in cleft patients. Furthermore, concurrent enrichment of the Wnt/β-catenin signaling pathway was observed in cleft patients. Our research findings provide compelling evidence for the evolutionary conserved similarities in pathways implicated in palatogenesis, as previously studied in murine models. Furthermore, we demonstrate the sustained maintenance of transcription factor expression differences even after the completion of palate development, thereby substantiating the existence of postnatal epigenetic influences on cleft pathogenesis. Indeed, Raposio *et al*.^30^ reported differences in the mitochondrial structure of the orbicularis oris muscle between the cleft side and the noncleft side in patients with unilateral cleft lip. This also provided a clue that alterations in the cleft side tissue exist even in patients with fully developed cleft lip, like our study. Remarkably, our study stands out as the pioneering investigation to comparatively analyze gene expression within the same individual, delineating spatial differences in gene expression by mucosal fusion. This unique approach not only advances our understanding of the intricate spatial regulation underlying palate development but also adds a novel dimension to the field of palatogenesis research by integrating intraindividual comparative analysis, thus enriching its scientific significance.

We conducted in vitro experiments to validate the RNA sequencing results we obtained previously. Notably, the EMT signaling pathway exhibited the most pronounced differential expression, and intriguingly, the top 10 differentially expressed pathways included the Wnt β-catenin, Shh, and TGF-β3 signaling pathways, which are recognized for their relevance to the EMT pathway. When the Wnt β-catenin pathway is activated, Wnts bind to the Frizzled protein on the cell membrane and LRP5/6, resulting in the translocation of β-catenin into the nucleus, where it becomes active^34^. The authors used Lrp6 to inhibit the Wnt β-catenin signal and examine the effects of inhibiting the Shh and TGF-β3 signals on EMT signaling. Our results revealed that shh and TGF-β3 also had an impact on some EMT assay outcomes, but they did not show consistent results. On the other hand, Lrp6, which is associated with Wnt/β-catenin signaling, consistently exhibited inhibitory effects in the EMT assay, indicating a closer association with EMT than the other two signals. Furthermore, in the RNA sequencing results, Wnt β-catenin signaling appeared as one of the top three differentially expressed pathways, which is consistent with these in vitro findings.

Our research also presents certain limitations. This study represents the inaugural effort to explore the transcriptomic differences between fused and unfused mucosa in patients with cleft palate, marking a pioneering approach that has not been undertaken previously. While there have been investigations into various transcriptomic alterations within the MEE in experimental animals, studies on human tissues are scarce, presenting a limitation. Previous research did not gather tissue samples directly related to MEE and actual palatal fusion, such as blood, lip tissue, or buccal tissue^25,35,36^. Our research stands out for utilizing mucosa from the palatal margin, a site crucially associated with palatal fusion and MEE. Furthermore, the RNA sequencing technique employed offers an impartial approach at the transcriptome level, making it a suitable method for providing evidence in groundbreaking research. To address these limitations in future studies, employing RNA sequencing analysis and protein metabolism level analysis in a broader population could reinforce our findings.

## Conclusion

Our study demonstrated differential gene expression in response to spatial variations in the presence of mucosal tissue within the same postnatal organism. Specifically, in our experiments investigating palatal fusion in animal models, the well-known EMT signal was found to be suppressed in cleft mucosa. Furthermore, the RNA sequencing data and in vitro experiments revealed an association with Wnt/β-catenin signaling, which is linked to EMT. This study provides evidence supporting existing findings in the field of postnatal genetics and contributes to the understanding of the significance of the EMT signaling pathway in palatal fusion in humans. Further research and animal model development are necessary to explore additional aspects of EMT-related palatal fusion.

## Ethical approval

Institutional Review Board at Keimyung University Dongsan Hospital (2020-07-034).

## Consent

Written informed consent was obtained from the patient for publication of this case report and accompanying images. A copy of the written consent is available for review by the Editor-in-Chief of this journal on request.

## Source of funding

This work was supported by a National Research Foundation of Korea (NRF) grant funded by the Korean government (MSIT) (No. 2021R1G1A1004556) and the Korea Medical Device Development Fund grant funded by the Korean government (the Ministry of Science and ICT, the Ministry of Trade, Industry and Energy, the Ministry of Health and Welfare, the Ministry of Food and Drug Safety) (Project Number: RS-2022-00140622).

## Author contribution

T.H.J.: wrote the draft of the manuscript, analyzed the data, and edited the figures; J.H.K.: analyzed the data; J.H.C. and J.H.K.: wrote sections of the manuscript; W.H.J.: conceived the study, analyzed the data, wrote sections of the manuscript, and edited the figures.

## Conflicts of interest statement

None of the authors have any financial arrangements or potential conflicts of interest related to this article.

## Research registration unique identifying number (UIN)

It is experimental study.

## Guarantor

Woonhyeok Jeong.

## Data availability statement

If there is request, data sharing is possible.

## Provenance and peer review

Not commissioned, externally peer-reviewed.

## References

[1] Hattori Y Pai BC Saito T . Long-term treatment outcome of patients with complete bilateral cleft lip and palate: a retrospective cohort study. Int J Surg 2023;109:1656–1667. 37073546 10.1097/JS9.0000000000000406 PMC10389451

[2] Woo AS . Evidence-based medicine: cleft palate. Plast Reconstr Surg 2017;139:191e–203e. 10.1097/PRS.0000000000002854 28027255

[3] Timbang MR Gharb BB Rampazzo A . A systematic review comparing furlow double-opposing Z-plasty and straight-line intravelar veloplasty methods of cleft palate repair. Plast Reconstr Surg 2014;134:1014–1022. 25347635 10.1097/PRS.0000000000000637

[4] Pang X Wang X Wang Y . Sox9CreER-mediated deletion of β-catenin in palatal mesenchyme results in delayed palatal elevation accompanied with repressed canonical Wnt signaling and reduced actin polymerization. Genesis 2021;59:e23441. 34390177 10.1002/dvg.23441

[5] Stanton E Sheridan S Urata M . From bedside to bench and back: advancing our understanding of the pathophysiology of cleft palate and implications for the future. Cleft Palate Craniofac J 2024;61:759–773. 36457208 10.1177/10556656221142098

[6] Jo T Choi K Choi J . The concordance of alveolar bone deficiency with severity of lip deformity in microform cleft lip. J Clin Med 2022;12:39. 36614840 10.3390/jcm12010039 PMC9821769

[7] Park MS Seo HJ Bae YC . Incidence of fistula after primary cleft palate repair: a 25-year assessment of one surgeon’s experience. Arch Plast Surg 2022;49:43–49. 35086308 10.5999/aps.2021.01396 PMC8795648

[8] Jung SE Ha S Koh KS . Clinical interventions and speech outcomes for individuals with submucous cleft palate. Arch Plast Surg 2020;47:542–550. 33238341 10.5999/aps.2020.00612 PMC7700856

[9] Hammond NL Dixon MJ . Revisiting the embryogenesis of lip and palate development. Oral Dis 2022;28:1306–1326. 35226783 10.1111/odi.14174 PMC10234451

[10] Nakajima A F. Shuler C Gulka AO D Hanai J . TGF-β Signaling and the epithelial-mesenchymal transition during palatal fusion. Int J Mol Sci 2018;19:3638. 30463190 10.3390/ijms19113638 PMC6274911

[11] Mukhopadhyay P Smolenkova I Seelan RS . Spatiotemporal expression and functional analysis of miRNA-22 in the developing secondary palate. Cleft Palate Craniofac J 2023;60:27–38. 34730446 10.1177/10556656211054004

[12] Shuler CF Guo Y Majumder A . Molecular and morphologic changes during the epithelial-mesenchymal transformation of palatal shelf medial edge epithelium in vitro. Int J Dev Biol 1991;35:463–472. 1801871

[13] Li R Chen Z Yu Q . The function and regulatory network of Pax9 gene in palate development. J Dent Res 2019;98:277–287. 30583699 10.1177/0022034518811861

[14] Auer PL Doerge RW . Statistical design and analysis of RNA sequencing data. Genetics 2010;185:405–416. 20439781 10.1534/genetics.110.114983 PMC2881125

[15] Subramanian A . Gene set enrichment analysis: a knowledge-based approach for interpreting genome-wide expression profiles. Proc Natl Acad Sci USA 2005;102:15545–15550. 16199517 10.1073/pnas.0506580102 PMC1239896

[16] Yoshioka H Suzuki A Iwaya C . Suppression of microRNA 124-3p and microRNA 340-5p ameliorates retinoic acid-induced cleft palate in mice. Development 2022;149:dev200476. 35420127 10.1242/dev.200476 PMC9148563

[17] Dobin A Davis CA Schlesinger F . STAR: ultrafast universal RNA-seq aligner. Bioinformatics 2013;29:15–21. 23104886 10.1093/bioinformatics/bts635 PMC3530905

[18] Li B Dewey CN . RSEM: accurate transcript quantification from RNA-Seq data with or without a reference genome. BMC Bioinformatics 2011;12:323. 21816040 10.1186/1471-2105-12-323 PMC3163565

[19] Bai Y Lu H Lin C . Sonic hedgehog-mediated epithelial-mesenchymal transition in renal tubulointerstitial fibrosis. Int J Mol Med 2016;37:1317–1327. 27035418 10.3892/ijmm.2016.2546

[20] Loh C-Y Chai JY Tang TF . The E-Cadherin and N-Cadherin switch in epithelial-to-mesenchymal transition: signaling, therapeutic implications, and challenges. Cells 2019;8:1118. 31547193 10.3390/cells8101118 PMC6830116

[21] Alade A Awotoye W Butali A . Genetic and epigenetic studies in non-syndromic oral clefts. Oral Dis 2022;28:1339–1350. 35122708 10.1111/odi.14146

[22] Sun Y . Genome-wide association study identifies a new susceptibility locus for cleft lip with or without a cleft palate. Nat Commun 2015;6:6414. 25775280 10.1038/ncomms7414

[23] Shaffer JR . Association of low-frequency genetic variants in regulatory regions with nonsyndromic orofacial clefts. Am J Med Genet A 2019;179:467–474. 30582786 10.1002/ajmg.a.61002 PMC6374160

[24] Mangold E . Genome-wide association study identifies two susceptibility loci for nonsyndromic cleft lip with or without cleft palate. Nat Genet 2010;42:24–26. 20023658 10.1038/ng.506

[25] Alvizi L Ke X Brito LA . Differential methylation is associated with non-syndromic cleft lip and palate and contributes to penetrance effects. Sci Rep 2017;7:2441. 28550290 10.1038/s41598-017-02721-0 PMC5446392

[26] Sharp GC Ho K Davies A . Distinct DNA methylation profiles in subtypes of orofacial cleft. Clin Epigenetics 2017;9:63. 28603561 10.1186/s13148-017-0362-2 PMC5465456

[27] Cáceres-Rojas G Salamanca C Krause BJ . Nonsyndromic orofacial clefts in Chile: LINE-1 methylation and MTHFR variants. Epigenomics 2020;12:1783–1791. 33147056 10.2217/epi-2020-0021

[28] Khan MFJ Little J Aleotti V . LINE-1 methylation in cleft lip tissues: Influence of infant MTHFR c.677C>T genotype. Oral Dis 2019;25 (6:1668–1671. 31161688 10.1111/odi.13136

[29] Razin A Cedar H . DNA methylation and gene expression. Microbiol Rev 1991;55:451–458. 1943996 10.1128/mr.55.3.451-458.1991 PMC372829

[30] Raposio E Bado M Verrina G . Mitochondrial activity of orbicularis oris muscle in unilateral cleft lip patients. Plast Reconstr Surg 1998;102:968–971. 9734410 10.1097/00006534-199809040-00005

[31] Raposio E Panarese P Santi P . Fetal unilateral cleft lip and palate: detection of enzymic anomalies in the amniotic fluid. Plast Reconstr Surg 1999;103:391–394. 9950523 10.1097/00006534-199902000-00005

[32] Hay ED . An overview of epithelio-mesenchymal transformation. Acta Anat (Basel) 1995;154:8–20. 8714286 10.1159/000147748

[33] Yu W Ruest LB Svoboda KK . Regulation of epithelial-mesenchymal transition in palatal fusion. Exp Biol Med (Maywood) 2009;234:483–491. 19234053 10.3181/0812-MR-365

[34] Liu J Xiao Q Xiao J . Wnt/β-catenin signalling: function, biological mechanisms, and therapeutic opportunities. Signal Transduct Target Ther 2022;7:3. 34980884 10.1038/s41392-021-00762-6 PMC8724284

[35] Machado RA Martelli-Junior H Reis SRA . Identification of novel variants in cleft palate-associated genes in brazilian patients with non-syndromic cleft palate only. Front Cell Dev Biol 2021;9:638522. 34307341 10.3389/fcell.2021.638522 PMC8297955

[36] Fu C Lou S Zhu G . Identification of new miRNA-mRNA networks in the development of non-syndromic cleft lip with or without cleft palate. Front Cell Dev Biol 2021;9:631057. 33732700 10.3389/fcell.2021.631057 PMC7957012

